# Altered activity patterns of transcription factors induced by endoplasmic reticulum stress

**DOI:** 10.1186/s12858-016-0060-2

**Published:** 2016-03-24

**Authors:** Sheena Jiang, Eric Zhang, Rachel Zhang, Xianqiang Li

**Affiliations:** Signosis Inc., 1700 Wyatt Drive, Suite #10-12, Santa Clara, CA 95054 USA; Saratoga High School, 20300 Herriman Ave, Saratoga, CA 95070 USA

**Keywords:** ER stress, UPR, TF, Plate array, Activation and Signal pathways

## Abstract

**Background:**

The endoplasmic-reticulum (ER) responds to the burden of unfolded proteins in its lumen by activating intracellular signal transduction pathways, also known as the unfolded protein response (UPR). Many signal transduction events and transcription factors have been demonstrated to be associated with ER stress. The process in which ER stress affects or interacts with other pathways is still a progressing topic that is not completely understood. Identifying new transcription factors associated with ER stress pathways provides a platform to comprehensively characterize mechanism and functionality of ER.

**Methods:**

We utilized a transcription factor (TF) activation plate array to profile the TF activities which were affected by ER stress induced by pharmacological agents, thapsigargin (TG) and tunicamycin (TM) at 1 h, 4 h, 8 h and 16 h respectively, in MiaPACA2 cells. The altered activity patterns were analyzed and validated using gel shift assays and cell-based luciferase reporter assay.

**Results:**

The study has not only confirmed previous findings, which the TFs including ATF4, ATF6, XBP, NFkB, CHOP and AP1, were activated by ER stress, but also found four newly discovered TFs, NFAT, TCF/LEF were activated, and PXR was repressed in response of ER stress. Different patterns of TF activities in MiaPaCa2 were demonstrated upon TM or TG treatment in the time course experiments. The altered activities of TFs were confirmed using gel shift assays and luciferase reporter vectors.

**Conclusion:**

This study utilized a TF activation array technology to identify four new TFs, HIF, NFAT, TCF/LEF and PXR that were changed in their activity as a result of ER stress induced by TG and TM. The TF activity patterns were demonstrated to be diverse in response to the duration of TG or TM treatment. These new findings will facilitate further unveiling the complex mechanisms of the ER stress process and associated diseases.

## Background

ER plays an important role in many biological functions such as folding and assembling the membrane and secreted proteins in eukaryotic cells [1]. Production process of these proteins in the lumen of the ER is believed to be led by the coordination between the extracellular and intracellular signals [2]. It has been previously reported [2–4] that, an imbalance between the protein-folding load and the capacity of the ER could happen due to either increase of protein-folding demand or disruption of protein-folding reactions, which will generate ER stress, and subsequently lead to accumulation of unfolded or misfolded proteins in the ER lumen. ER stress simultaneously activates Unfolded Protein Response (UPR) to reduce protein synthesis, degrade misfolded proteins, and produce molecular chaperones. The growing evidence suggests that ER stress (UPR) is an intricate molecular process, interacting with oxidative stress [5], Ca^2+^ signal response [6], and the inflammatory response and other signal pathways. In addition, ER stress is associated with a variety of diseases caused by the accumulation of aggregated proteins such as neurodegenerative diseases and diabetes [7].

It is well-known that in mammalian cells, the ER stress activates three distinct ER-localized transmembrane proteins, inositol-requiring enzyme 1 (IRE1), pancreatic ER kinase (PERK), and activating transcription factor 6 (ATF6) [8, 6]. The timing and duration of activation of these three proteins may be different from the previous speculation of parallel responses [9]. For examples, the activation of PERK could inhibit the global protein translation through phosphorylation of eIF2α, and a translational increase in the transcription factor ATF4 would promote UPR-specific gene expression [10, 11], and IRE1 would lead to the generation of a more potent form of XBP1 mRNA splicing version [12]. Furthermore, ATF6, an ER membrane transcription factor [13], undergoes proteolysis to release its cytoplasmic transactivation domain to become active [9]. Therefore, PERK, IRE1, and ATF6 are ultimately responsible for the activation of a set of transcription factors through a complicated and nonparallel process.

Cells respond to ER stress by inducing gene expression. Consequently, a signal transduced from the ER to the nucleus is required to activate transcription in response to the ER Stress. Activated TFs as the endpoints of the signal transduction pathway directly regulates the final gene expression in the nucleus. Therefore, TF activation is a measure of the effectiveness of ER stress in activating the three proteins mentioned above in the cells. A number of transcription factors have been found to take part in ER stress response, such as ATF6 [13], XBP1 [12], ATF4 [9], NFkB [14], AP1 [14], SREBP and CHOP (C⁄EBP homology) [15]. The activation of these TFs is believed to be associated with ER stress but through different mechanisms. The intrinsic ribonuclease activity of IRE1 also results in production and activation of XBP-1, inducing expression of genes involved in restoring protein folding or degrading unfolded proteins [12]. ATF4 is translationally up-regulated by eIF2a-mediated translational attenuation and PERK/eIF2α ∼ P/ATF4 pathway is required not only for translational control, but also for activation of ATF6 [9] and CHOP and their target genes. Oligomerized Ire1 binds to TRAF2, TNF receptor associated factors, that activate NF-κB and c-Jun (AP-1), leading to expression of a set of genesassociated with host defense or alarm [14]. In addition, the different transcription factors may display different response time patterns during ER stress process and variable pathways.

Pharmacological agents are commonly used to treat cells to elevate unfolded proteins in most studies of ER stress, including dithiothreitol (DTT), which disrupts or prevents protein disulfide bonding; thapsigargin (TG), an inhibitor of the ER Ca2 dependent ATPase; or tunicamycin (TM), an inhibitor of protein glycosylation of newly synthesized proteins [16]. However, the concentration and duration of treatment vary from system to system. Typically only a few hours are sufficient to induce ER stress while a longer exposure often lead ER stress-mediated cell to death. Previous studies have indicated that three ER transmembrane components, IRE1, PERK and ATF6, displayed distinct sensitivities toward different forms of ER stress induced by these three agents, but it is not clear how ER stress is affected in downstream pathways and transcriptional regulation.

In this study, we employed a TF activation profiling array to systematically monitor ER stress-induced TF activity patterns with 1 h, 4 h, 8 h and 16 h of TM and TG treatment in pancreatic tumor cell MiaPaCa2, since this cell line has demonstrated a globally compromised ability to regulate the unfolded protein response and it has been widely used for studying ER stress process [17–19]. With the plate array assay, the activities of 48 TFs can be elucidated in a single experiment. Through a comparative study, it was observed that the activities of ATF4 and ATF6, XBP1, CHOP, AP1, NFkB, NFAT, TCF/LEF and HIF increased, while the activity of PXR decreased to different extents in response to TM and TG treatment. To our knowledge, the activation of NFAT, TCF/LEF, HIF and PXR under ER stress was observed for the first time. The altered TFs were further confirmed by conventional gel shift assays and luciferase reporter assays. Different patterns of TF activities in MiaPaCa2 were exhibited in response to different TM or TG treatment time, which may help to unveil the complicated mechanism of ER stress process.

## Methods

### Cell culture and nuclear extraction

MiaPaCa2 cells were seeded in 10 cm^2^ culture plates in ATCC-formulated Dulbecco’s Modified Eagle’s Medium (ATCC), supplemented with 10 % FBS, 1 % nonessential minimal amino acids and 100 U/ml penicillin, 0.1 mg/ml streptomycin. The cells (about 80 % confluent) were then treated with 200nM TG and 10ug/ml TM for 1 h, 4 h, 8 h and 16 h respectively. Untreated cells were used as negative controls. Nuclear extracts were prepared with the nuclear extract kit (Signosis, Inc.) according to the user manual. The cells were washed twice in phosphate-buffered saline (PBS) and lysed on ice for 10 min in the extraction buffer I with gently shaking, and then were collected from the plates, and centrifuged at 15,000 rpm for 3 min at 4 °C. The supernatant (cytoplasmic fraction) was discarded; the pellet was then resuspended in 250 μl of extraction buffer II and incubated on ice for 2 h with gently shaking. After the mixture was centrifuged at 15,000 rpm for 5 min at 4 °C, the supernatant containing nuclear protein was collected and ready for assays. Protein concentrations were determined by the Bradford assay (Bio-Rad).

### TF activation profiling analysis

Each array assay was performed following the procedure described in the TF activation profiling plate array kit user manual (Signosis, Inc). 10 ug of nuclear extract was first incubated with the biotin labeled probe mix at room temperature for 30 min. The activated TFs were bound to the corresponding DNA binding probes. After the protein/DNA complexes were isolated from unbound probes, the bound probes were eluted and hybridized with the plate pre-coated with the capture oligos. The captured biotin-labeled probes were then detected with Streptavidin–HRP and subsequently measured with the chemiluminescent plate reader (Veritas microplate luminometer).

### Gel shift assay

The samples with 8 h of TG and TM treatment were chosen for gel shift assay analysis with EMSA kits (Signosis Inc). The TF DNA binding probe sequences are listed below.

1. ATF: CTGTCATGACGTCAAAAGTCG
2. NFkB: AGTTGAGGGGACTTTCCCAGGC
3. NFAT: ACGCCCAAAGAGGAAAATTTGTTTCATACA
4. AP1: CGCTTGATGACTCAGCCGGAA
5. CHOP: TTGCGGAGGATTGCGTTGACGA
6. TCF/LEF: ACGTTACTTTGATCTGATCAGGGC
7. XBP1: GATCTCCTAGCAACAGATGCGTCATCTC
8. HIF: GTGACTACGTGCTGCCTAG

The sequences that we used as probes for gel shift assay are identical to those we used as the probe mix for TF activation profiling array assay. 5ug nuclear extracts were incubated with 1× binding buffer and biotin-labeled probe for 30 min at room temperature to form protein/DNA complexes. The samples were then electrophoresed on a 6 % polyacrylamide gel in 0.5 % TBE at 120 V for 45 min and then transferred onto a nylon membrane in 0.5 % TBE at 300 mA for 1 h. After transfer and UV cross-linking, the membrane was detected with Streptavidin–HRP. The image was acquired using a FluorChem imager (Alpha Innotech Corp).

### Luciferase reporter assay

Luciferase reporter assay was carried out following the procedure in Luciferase reporter assay user manual (Signosis, Inc.). The reporter vectors contain 4 repeats of the corresponding DNA binding sequences shown in gel shift assay section. In order to distinguish ATF4 and ATF6 activation, we cloned the reporter vectors for ATF4 and ATF6 with 5 repeats of specific consensus sequences for ATF4 and ATF6 respectively, TGACGTAAG [20] for ATF4 and TGACGTGG [21] for ATF6. The cells were first transfected with luciferase reporter vectors (Signosis Inc) for 16 h with Fugene 6 (Promega) in a 96-well plate, and then treated without or with 200nM TG and 10ug/ml TM for 6 h, 8 h and 16 h respectively. After removing the culture media and rinsing the cells twice with PBS, 200 μl of 1× cell lysis buffer was added to lyse the cells. After dislodging the cells by scraping them off from the plate, we transferred the cells to a 1.5-ml microcentrifuge tube before being centrifuged at 14,000 rpm at room temperature for 1 min to remove cellular debris. 10 μl of the cell extract was mixed with 50 μl of substrate (Signosis Inc), and luminescence was measured using a luminometer.

### Statistical analyses

Data were analyzed by a method of two-sided and unpaired *t*-test using GraphPad Prism 6.0 software. The mean ± SD of multiple independent experiments were shown in data analysis. A *p* value of <0.05* would be considered significant, *p* < 0.01** very significant, and *p* < 0.001*** highly significant.

## Results and discussion

**Fig. 1:**
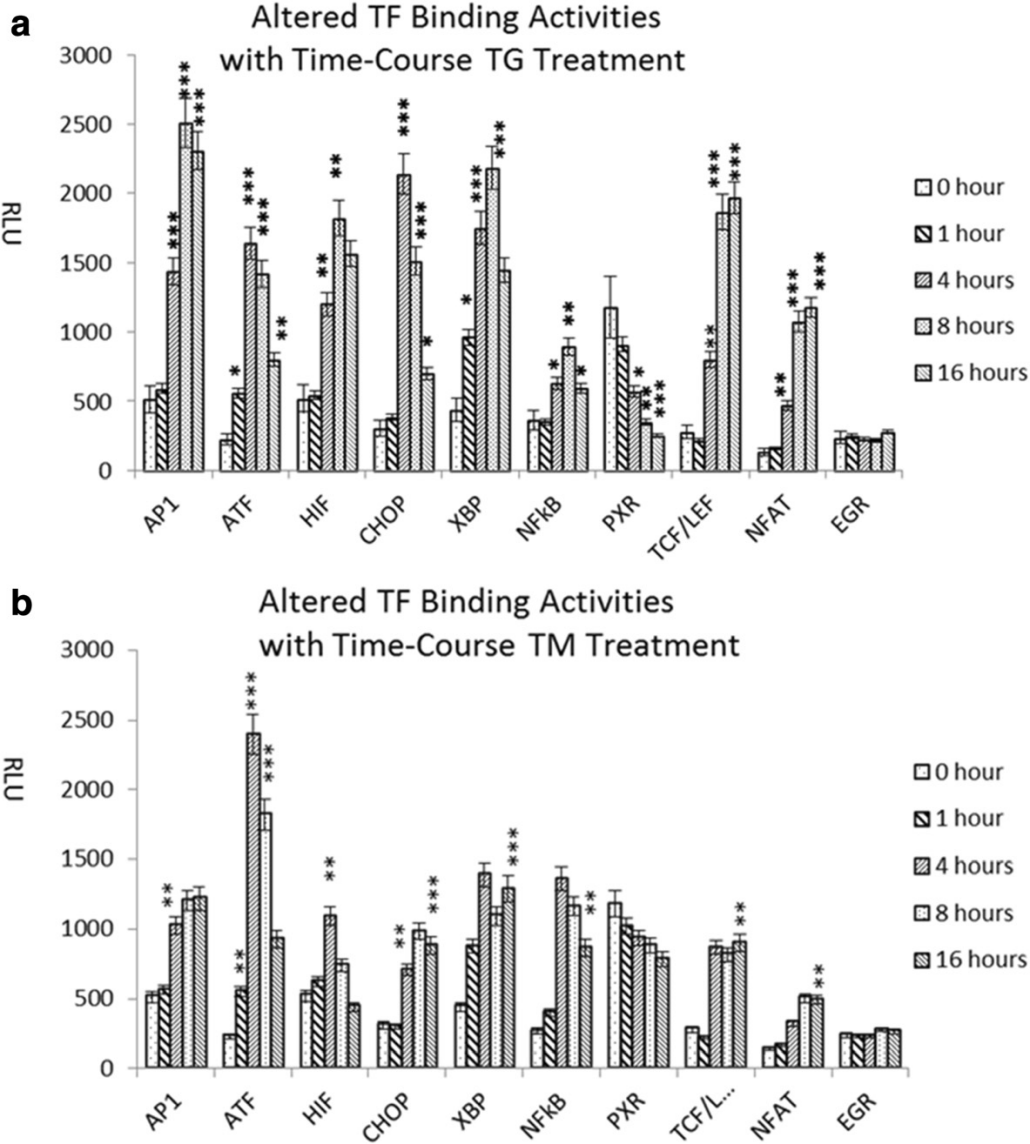
Plate array analysis of 48 TFs in MiaPAC2 cells treated without or with TG/TM treatment respectively. After 1 h, 4 h, 8 h and 16 h of treatment, the cells were subjected to nuclear extraction. The nuclear extracts were then used for TF activation plate assay. The data from control sample (without treatment), TG treated and TM treated samples were compared. Data were obtained from three independent experiments, *P < 0.05, **P < 0.01, *** *P* < 0.001; (**a**): TF activation DNA binding assay with TG treatment; (**b**). TF activation DNA binding assay with TM treatment

To examine TF activation patterns induced by ER stress, MiaPAC2 cells were treated with or without TG or TM for 1 h, 4 h, 8 h and 16 h prior to preparation of nuclear extracts for analysis with the TF activation profiling plate array I with slight modification (Table 1). The TFs were selected based on their important biological functions in crucial signal pathways which may associate with ER stress. The nuclear extracts were mixed with a biotin-labeled pool of DNA probe mix that correspond specifically to 48 TF response elements. After the probes were incubated with nuclear extracts, the complexes of TFs and probes were separated from the free probes. Through elution of bound probes, the composition and quantity of the bound probes were then determined using a plate array, which contained the pre-coated capture oligos in a 96-well white plate according to the position of the individual TFs indicated in Table 1, therefore, the plate would hybridize with any labeled probe that was present. After the hybridized signals were detected with a Streptavidin–HRP and HRP substrate, the resulting chemiluminescence was measured by a plate reader. The evolution of TF activity pattern in response to ER stress process was examined in a chronological sequence. The ATF and XBP1 activities were shown to increase significantly after only 1 h of TG and TM treatment. The activities of CHOP, AP1, NFkB, NFAT, TCF/LEF and HIF showed significant increases after 4 h of TG and TM treatment. All of TF activities reached to peak upon 8 h of TG and TM treatment. After 16 h treatment, only NFAT and TCF/LEF activities remained the same level as 8 h treatment, and the other TFs all decreased slightly (Fig. 1). In addition, we identified that the activity of PXR decreased significantly after 4 h of TG treatment but only slightly decreased in TM-treated cells. Furthermore, HIF, TCF/LEF, NFAT and PXR were observed to be ER stress responsive TFs for the first time.

**Table 1:** The diagram of TF Activation Plate Array I (revised). 48 TFs are included, locating in the column 1–6 and column 7–12 respectively

	1	2	3	4	5	6	7	8	9	10	11	12
A	AP1	CDP	GATA	XBP	Pit	Stat3	AP1	CDP	GATA	XBP	Pit	Stat3
B	AP2	CREB	GR/PR	NFAT	PPAR	Stat4	AP2	CREB	GR/PR	NFAT	PPAR	Stat4
C	AR	E2F-1	HIF	CHOP	PXR	Stat5	AR	E2F-1	HIF	CHOP	PXR	Stat5
D	ATF	EGR	HNF4	NFkB	SMAD	Stat6	ATF	EGR	HNF4	NFkB	SMAD	Stat6
E	Brn-3	ER	IRF	Oct-4	Sp1	TCF/LEF	Bm-3	ER	IRF	Oct-4	Sp1	TCF/LEF
F	C\EBP	Ets	MEF4	p53	SRF	TR	C\EBP	Ets	MEF2	p53	SRF	TR
G	CAR	FAST-1	Myb	Pax-5	SATB1	YY1	CAR	FAST-1	Myb	Pax-5	SATB1	YY1
H	CBF	GAS/ISRE	Myc-Max	Pbx1	Stat1	TFIID	CBF	GAS/ISRE	Myc-Max	Pbx1	Stat1	TFIID

**Fig. 2:**
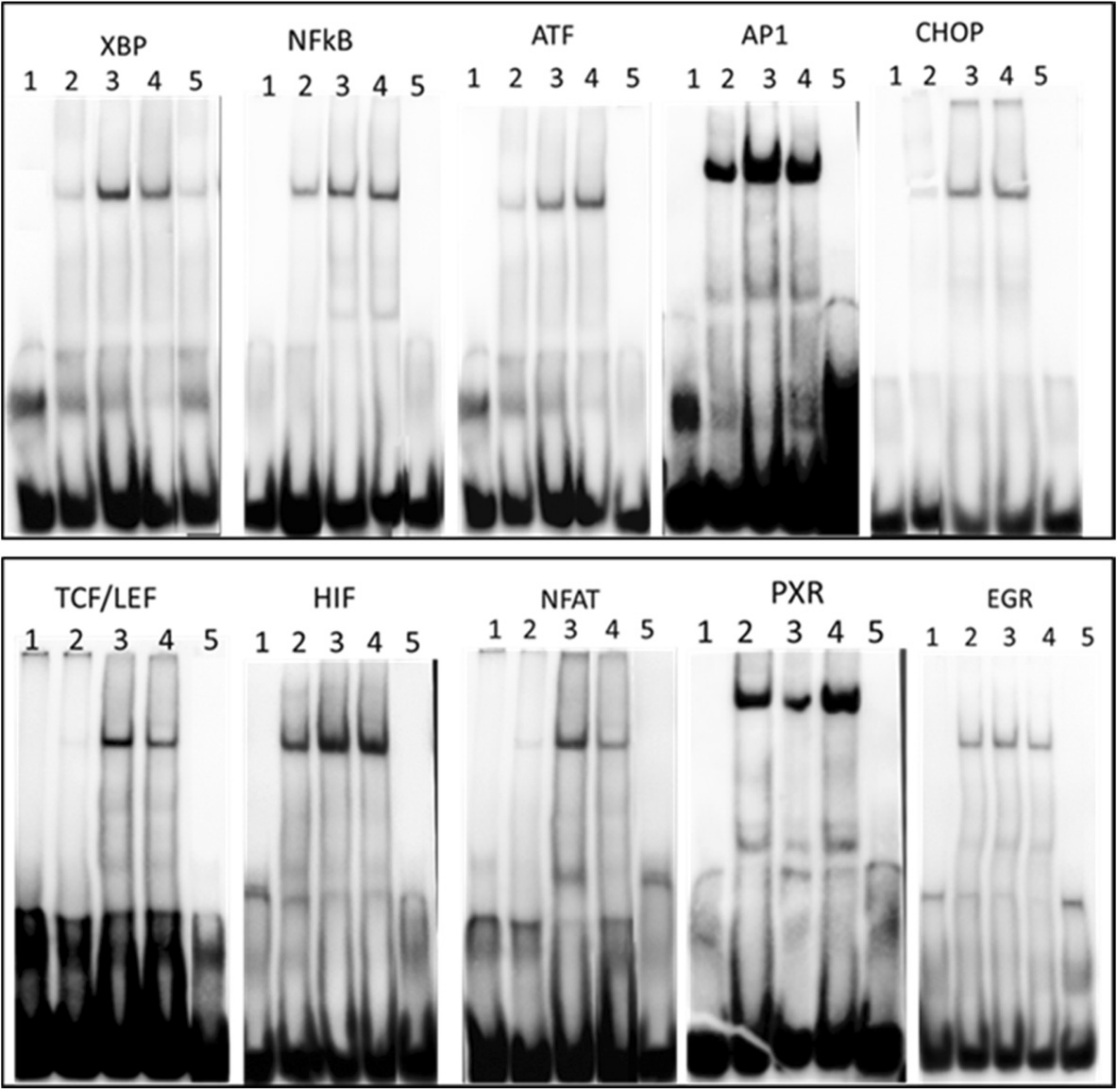
Nuclear extracts with 8 h of treatment were subjected to EMSA assay with different probes. **a**: TFs, XBP, NFkB, ATF, AP1 and CHOP, were reported to be associated with ER stress previously. **b**: TFs, TCF/LEF, HIF, NFAT and PXR, were the first time reported to be associated with ER stress in this study. EGR was used as a negative control. 1. Free probe only. 2. Without treatment; 3. TG treatment; 4. TM treatment; 5. Cold probe competition

In order to validate the plate array results, the samples with optimal 8 h of TG and TM treatment were used for gel shift assay. As shown in Fig. 2, both TG- and TM-activated ATF, XBP1, CHOP, AP1, NFkB, NFAT and HIF were able to be confirmed with gel shift assays. The decrease of PXR in DNA binding activity in TG-treated cells was also confirmed with the gel shift assay but the slight change in the activity of PXR in TM-treated cells identified by the plate array was not detectable with the gel shift assay. As the gel shift assay is considered to be a gold standard in analysis of DNA binding activities of TFs, we concluded that the activities of PXR decreased in TG-treated but not TM-treated cells. Furthermore, we introduced EGR as a control in gel shift assay. The array data showed no change of ERG in either TG- or TM- treated cells as compared to the untreated MiaPAC2 cells. The gel shift assay showed no difference in EGR between treated and untreated cells (data not shown). Through both the array and gel shift assays, we confirmed that ATF, XBP1, CHOP, AP1, NFkB, TCF/LEF, NFAT and HIF are indeed activated by TG and TM. The activity of PXR was down regulated by TG but not by TM.

**Fig. 3:**
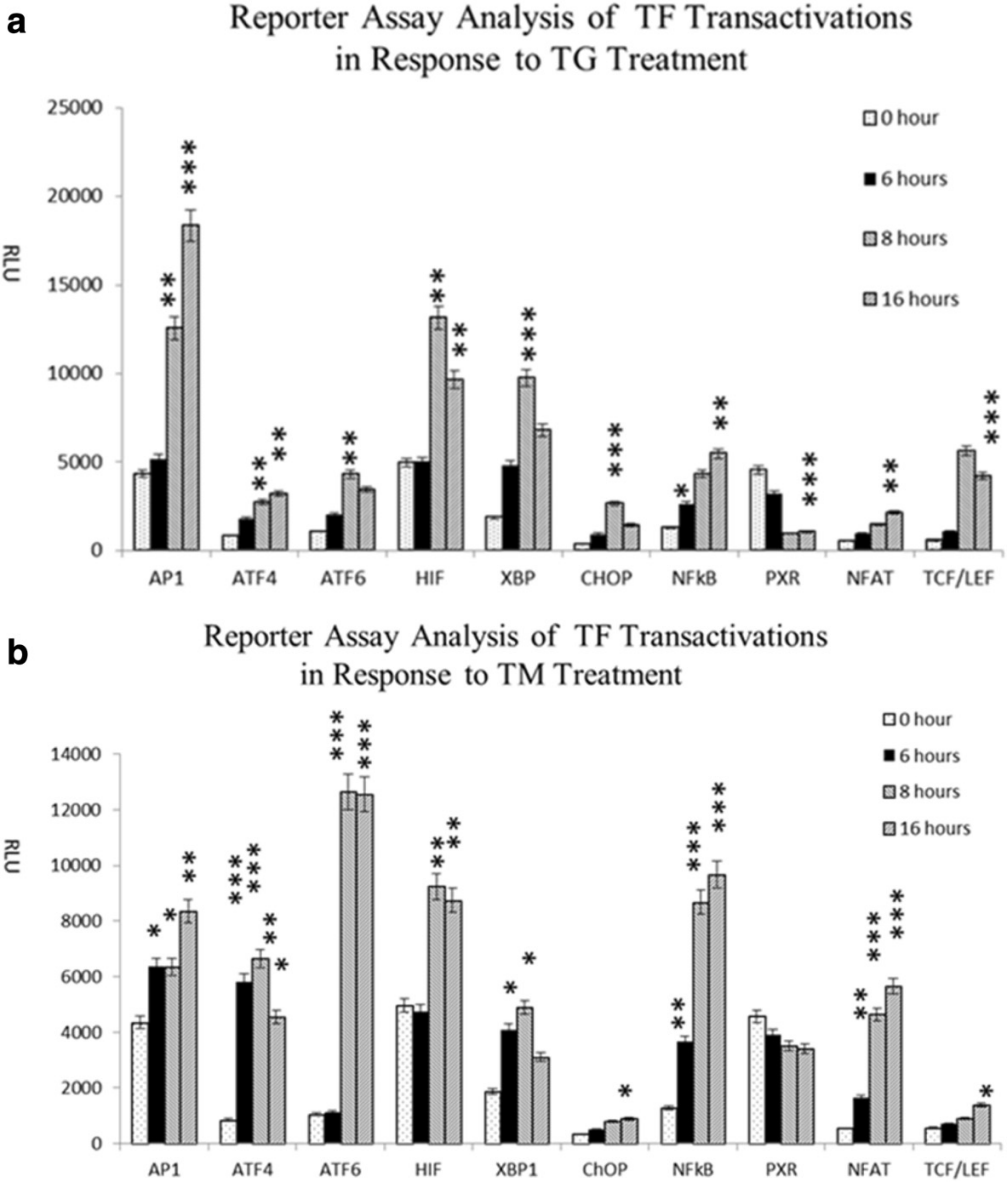
Transactivation of TFs in response to TG and TM treatment. The cells were transfected with different reporter vectors for 16 h, and treated with TG or TM respectively for 0 h (no treatment), 6 h, 8 h and 16 h The cells then were lysed and subjected to luciferase assay. Data were obtained from three independent experiments, *P < 0.05, **P < 0.01, *** *P* < 0.001; (**a**): Reporter assay with TG treatment; (**b**): Reporter assay with TM treatment

In order to investigate whether the activation of these TFs can be quantitatively monitored with luciferase reporter assays, we employed a set of reporter vectors corresponding to these TFs to transfect into MiaPAC2 cells. The consensus sequence of ATF probe in the TF activation plate array assay and gel shift assay are for ATF family but cannot distinguish ATF family members, ATF4 and ATF6. We designed and cloned ATF4 and ATF6 reporter vectors with specific ATF4 and ATF6 DNA binding sequences respectively. After transfection of the vectors into the cells, the cells were treated with TG and TM treatment for 6 h, 8 h and 16 h before their luciferase activity was measured. We confirmed the activation of ATF4, ATF6, XBP1, CHOP, AP1, NFkB, TCF/LEF, NFAT and HIF by TG and TM treatment, and repression of PXR by TG only but not by TM. The activation of ATF4 and XBP1 was observed to occur at the earlier stage during ER stress process. In addition, TG was shown to be a stronger inducer for CHOP, XBP, AP1, TCF/LEF, and PXR, whereas TM-activated ATF4, ATF6 and NFkB were shown to much more effective than TG. HIF responded equivalently to both TM and TG treatments (Fig. 3).

A possible mechanism underlying alteration of newly identified NFAT, HIF, TCF/LEF and PXR activities during ER stress process is presented here for further discussion. TM blocks the initial step of glycoprotein biosynthesis in the ER. Thus, treatment of TM causes accumulation of unfolded glycoproteins in the ER, effectively triggers eIF2α/ATF4 pathway and activates ATF4. ATF4 has demonstrated to be an early activated TF during early ER stress process and is the master regulator that plays a crucial role in the adaptation to stresses by regulating the transcription of many genes, such as CHOP and ATF6. These ATF4 target genes are themselves transcription factors that regulate the expression of a set of stress-induced target genes and amplify the signals by triggering other signaling pathways, such as inflammation and hypoxia via activating NFkB and HIF. Activation of these multiple TFs by the ER (UPR) may result in a complicated pattern of gene regulation through not only by target gene regulation but also by protein/protein interaction. It agrees with the observation from one of our previous studies that, the altered activities of TFs can be induced either by over expression TF or by interaction of TF with other proteins [22]. In addition, TG that can generate Cadysregulation and induce ER stress may result in significant increases in cytosolic Ca^2^. Ca^2^ disequilibrium releases beta-catenin from the plasma membrane, which subsequently leads to the accumulation of beta-catenin in the cytoplasm and formation of beta-catenin/TCF/LEF complex. This complex further translocates to the nucleus where it activates transcription [23]. Increased Ca^2^ concentration in the cytoplasm could also directly activate NFAT. ER stress has been reported to lead to the phosphorylation of HIF-1α, which might result in an increase in the activity of HIF-1α [24, 25]. PXR is a nuclear receptor recognized as a major regulator of xenobiotic metabolism and drug metabolism by regulating CYP3A4 [26]. The expression of PXR has been reported to be suppressed during ER stress by down-regulating HNF4 and up-regulating liver-enriched inhibitory protein (LIP) with TG treatment. The decrease in DNA binding activity of TG-induced PXR discovered in this study may be due to the pathway interactions with HNF4, ATF and CHOP [27]. With the array assay the different TF activity patterns were displayed in response to different ER stress pathways. Formation of homo- and heterodimers among these TFs families may build an integrated transcription factor network that determines precisely the initiation, magnitude, and length of the cellular response to ER stress in a fine-tuned and coordinated way. In spite of the exact mechanism how ER stress is regulated by TFs still remains not fully clear, the new findings in this study offer clues to dissect the cellular response to ER stress signaling pathways.

## Conclusion

We used TF activation plate array to profile the TF activities of TFs and reported four newly identified TFs whose activities were altered in response to ER stress. The activity patterns were shown to be distinctive with the different ER signal pathways.

## Abbreviations

AP1: activating protein
ATF: activating transcription factor
CHOP: DNA damage inducible transcript 3
ER: endoplasmic reticulum
HIF: hypoxia inducible factor
NFkB: nuclear factor of kappa light polypeptide gene enhancer in B-cells
PXR: pregnane X receptor
TCF/LEF: T-cell factor/lymphoid enhancer factor
TF: transcription factor
TG: thapsigargin
TM: tunicamycin
UPR: unfolded protein response
XBP: X-box binding protein
